# Case Report: A Presentation of Early-Onset Immune-Mediated Bullous Pemphigoid in a Patient with Urothelial Cancer

**DOI:** 10.3390/curroncol30090566

**Published:** 2023-08-23

**Authors:** Josep Sabaté Ortega, Roser Fort Culillas, Marina Escoda Garcia, Carmen Amalia Vásquez-Dongo, Núria Sala González

**Affiliations:** 1 Oncology Department, Catalan Institute of Oncology, Hospital Universitari Doctor Josep Trueta, 17007 Girona, Spain; rfort@iconcologia.net; 2 Dermatology Department, Hospital Universitari Doctor Josep Trueta, 17007 Girona, Spain; mescoda.girona.ics@gencat.cat; 3 Pathology Department, Hospital Universitari Doctor Josep Trueta, 17007 Girona, Spain; cavasquez.girona.ics@gencat.cat

**Keywords:** immune checkpoint inhibitors, immune-related adverse events, bullous pemphigoid, atezolizumab

## Abstract

Cutaneous immune-related adverse events (cirAEs) are the most common side effects of immune checkpoint inhibitor (ICI) therapy (30–50% for all grades). The vast majority of them are low or mild and can be treated without ICI interruption. Autoimmune blistering disorders, such as immune-mediated bullous pemphigoid (IBP), are rare (<1%) but potentially serious conditions that must be early detected. The onset generally occurs within the first months of the treatment, and it appears to be more common with antiprogrammed death-1 or antiprogrammed ligand 1 (anti-PD1/PDL1) than with anticytotoxic T-lymphocyte-associated protein 4 (anti-CTLA4). We present a case of a three-day severe IBP onset after receiving the first cycle of atezolizumab. This exceptional early presentation could suggest the presence of some predisposing condition and demonstrates the need to better understand predictive toxicity-related biomarkers in candidate patients for immunotherapy.

## 1. Introduction

Immune checkpoint inhibitors (ICIs) are a novel class of immunotherapy drugs that have emerged as the mainstay of cancer treatment over the past few years. ICIs block the inhibitory signals that are generated by tumor cells inhibiting the immune system, which prevents its detection and destruction [1]. The augmented immune response enabled by these agents has led to immune-related adverse events (irAEs), reported in 30–50% of patients receiving ICIs. High-grade irAEs are less frequent (10–15%) [2]. Up to 1.3% of patients have experienced irAE fatalities [3]. The likelihood of irAEs increases over time, but they often appear within a year of treatment [4]. Effectively addressing the management of immune-related adverse events (irAEs) requires a comprehensive and diverse approach, involving active participation and collaboration from patients, medical providers, and institutions across multiple levels. Patients and the medical community need institutional safeguards in place to promote swift recognition and encourage rapid detection, assessment, and treatment of immune-related toxicities [5].

The range of organ systems affected by irAEs is very broad. Therefore, toxicities of varying frequency and severity can affect almost any organ [6]. Skin rashes, hepatitis, thyroiditis, hypohysitis, myocarditis, pericarditis, arthritis, colitis, uveitis, or pneumonitis are the common toxicities of ICIs [7]. The most frequent irAEs are dermatotoxicities, and they can occur in up to 30–50% of patients receiving ICIs [8]. Pruritus, exanthems, vitiligo, and lichenoid responses are some of the most typical dermatotoxicities observed in these patients [9].

BP is a rare autoimmune subepidermal blistering disease characterized by the development of tense bullae [10]. Mucosal involvement is usually not reported [11]. The onset is usually within five to nine weeks [12,13,14].

Perioperative ICIs have provided persuading efficacy along with low toxicity in the neoadjuvant setting. Atezolizumab is a humanized IgG1 monocolonal antibody that targets PDL-1, and it has been approved for the treatment of locally advanced and metastatic urothelial cancer after plantinum-containing chemotherapy and many other cancer types. For muscle-invasive bladder cancer (MIBC), the results regarding tolerability and toxicity have been even better than chemotherapy and have proved to be a potential modern therapeutic intervention for refractory non-MIBC. Due to these reasons, ICIs are more frequently incorporated into the treatment regimens for metastatic urothelial cancers [15,16].

Combinations currently employed with atezolizumab include radiotherapy, chemotherapy, epigenetic modulators, angiogenesis inhibitors, other immune checkpoint inhibitors, and transplantation of fecal microbiota [17]. A combination therapy used for invasive bladder carcinoma is BPR-ART (bladder preservation therapy in combination with atezolizumab and radiation therapy). This combination was focused on grades T2–T3 cancer (16.2 Gy to the whole bladder and 41.4 Gy component to the smaller pelvic field) and atezolizumab to the patient at 3-week intervals. Compared to monotherapy, this combination demonstrated high pathological complete response (pCR) rates for high-risk patients (older patients and those having a higher expression of PD-L1) [18]. In patients with locally advanced/metastatic UC, the combination of atezolizumab with front-line platinum-based chemotherapy (using the chemotherapy agents gemcitabine and cisplatin or carboplatin) showed a potentially improved median PFS (8.2 months). The hazard ratio was 0.82 (*p* = 0.007), and the objective response rate was 47%, compared to the 23% objective response rate observed with atezolizumab monotherapy. The overall response rate in the combination therapy was 13%, whereas monotherapy was 6%. Patients treated with the combination therapy had better survival rates and lower risks of hazards than those treated with monotherapy [19].

A new horizon is opened by incorporating molecular classification into the neoadjuvant context. Immunogenome profiles and tumor subtypes can be used to drive personalized treatment strategies that could lead to better patient outcomes. PD-L1 immunohistochemistry, high tumor mutation load (TMB), circulating tumor DNA (ctDNA), DNA damage response gene alterations (DDR), and tumor-infiltrating lymphocytes (TILs) appear to be prognostic indicators associated with the response to neoadjuvant immunotherapy invasive bladder carcinoma. The lack of comprehensive long-term survival data for patients exposed to ICIs underscores the need for additional research to substantiate the apparent survival benefits. This transition to a more personalized treatment approach would be facilitated through the integration of new biomarkers and molecular changes, thereby reshaping the existing standard of care [15,16,20].

The atezolizumab toxicity profile is relatively favorable when compared to that of conservative cytotoxic chemotherapy, particularly in bladder carcinoma. Patients treated with atezolizumab demonstrated a lower rate of immune-related toxicity withdrawal [13] and an observed death rate of 0.17%. The meta-analysis focused on 3266 patients, revealing that the total risk of any grade adverse event was 69%. Grade 3 or more severe adverse events (AEs), however, occurred in 13% of the participants [21]. We present a case of early-onset grade 3 IBP induced by atezolizumab.

## 2. Presentation of the Case

A 79-year-old male with no smoking history presented with recurrent gross hematuria. His medical history included hypertension, diabetes, dyslipidemia, and obesity, while there was no evidence of autoimmune or skin disorders. Ultrasound imaging revealed a solid mass with low echogenicity in the bladder, and the computed tomography (CT) scan confirmed a large solid mass in the lower right anterior bladder wall measuring approximately 5.5 × 4.7 cm with involvement of the urethral meatus. The bladder tumor was removed transurethrally from the patient. He was diagnosed with locally advanced bladder cancer stage cT2N0M0 (AJCC 8th edition). Histopathological findings revealed poorly differentiated invasive urothelial carcinoma (UC) with muscularis propria invasion.

From August 2020 to November 2020, he received neoadjuvant cisplatin/gemcitabine chemotherapy for four cycles with good tolerance. After that, he underwent laparoscopic radical cystectomy with the Bricker-type urinary diversion in November 2021.

**Figure 1:**
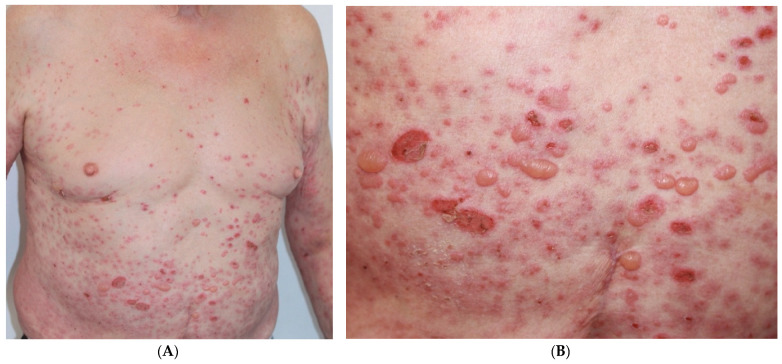
(**A**). Vesicular-bullous lesions and crusts on erythematous skin and skin of normal color on trunk and upper extremities. (**B**). Blisters and tense vesicles with serous content along with erosions and crusts.

In April 2021, a PET/CT scan revealed locoregional lymph node recurrence and a 30 × 20 solid mass in the surgical bed infiltrating the ischiopubic muscle. He started first-line treatment with concomitant radiotherapy (46 Gy in 23 fractions) and weekly cisplatin at 30 mg/m^2^ until July 2021. A partial response was achieved at the pelvic ganglion level, leading to the decision of stopping treatment and starting follow-up. In November 2021, the CT scan demonstrated local disease progression, and the patient started atezolizumab 1200 mg in 3 weekly cycles as second-line therapy in January 2022. Three days after the first administration of treatment, he presented with a severe, widespread, shadowy vesicular-bullous lesions, generalized tense, without palmoplantar involvement. Some lesions had ruptured leaving erosive areas. Along with that, crusts with serous content on the erythematous skin on the trunk and upper extremities with no mucosal damage were also observed (Figure 1).

**Figure 2:**
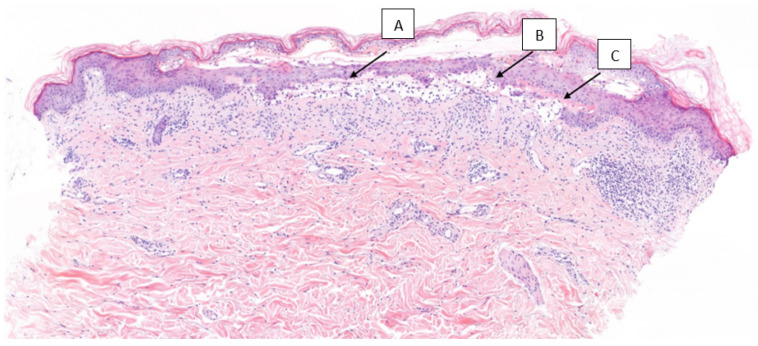
(**A**). Cutaneous punch biopsy showing subepidermal blisters with mixed inflammatory infiltrate inside including eosinophils. (**B**). A re-epithelization zone is observed in the upper layers of the dermis. (**C**). A mild perivascular chronic dermal infiltrate with eosinophils present.

A cutaneous punch biopsy showed subepidermal blisters with a mixed inflammatory infiltrate of eosinophils. A re-epithelization zone was observed in the upper layers of the dermis, and a mild perivascular chronic dermal infiltrate with eosinophils was observed. Direct immunofluorescence showed IgG and C3 deposits along the dermo–epidermal junction. To rule out potential underlying causes, the patient’s serologies for hepatitis B, hepatitis C, and HIV were also performed, and the results were negative. Moreover, the patient was screened for any autoimmune disorders by evaluating antinuclear antibodies (ANAs), which revealed a titer of less than 1/80. The enzyme-linked immunosorbent assay (ELISA) revealed a positive BP180 autoantibody (Figure 2) and antidesmosome BP230 antibodies (value of 0.23). The presence of these antibodies supported the diagnosis of BP. 

The incidence of BP revealed a possible grade 3 atezolizumab-linked dermatological irAE, and therefore the drug was stopped and corticosteroid therapy (oral prednisone) at 1 mg/kg/day was started. The patient underwent a gradual tapering off of oral corticosteroids over a period of 9 weeks. Additionally, topical therapy of potassium permanganate (1/10,000) was applied to the erosive and humid areas, followed by the application of beclometasone dipropionate and gentamicine cream every 12 h. This treatment approach led to a slow but complete remission of the cutaneous lesions within a few weeks. No relapses occurred. In February 2022, a CT scan revealed lesion progression toward the pelvic region. The treatment was discontinued due to treatment-limiting toxicity.

## 3. Discussion

Dermatologic toxicities are the most common irAEs. Immunobullous eruptions occur in approximately 1% of patients receiving PD-1/PDL-1 antibodies, but it is not typically associated with anti-CTLA-4 monotherapy, suggesting they may be a class effect of anti-PD-1/PD-L1 therapy [22]. They may occur as a consequence of immune system reactivation due to the blockage of PD-1/PD-L1, pre-existing organ-specific autoimmunity, environmental triggers, and a shared antigen between host cells and cancerous cells. Tumor-associated antigens (TAAs) encompass proteins, glycoproteins, polysaccharides, and other molecules, which can be present at lower levels in healthy host cells [21,23].

Studies have indicated that monotherapy with PD-1/PD-L1 inhibitors is associated with a higher incidence of pruritus and autoimmune bullous skin disorders [14,24]. BP is an autoimmune blistering disorder that is caused by autoantibodies against the hemidesmosomal proteins BP180 and BP230 at the skin basement membrane [25]. Over 90% of BP patients have IgG autoantibodies that target the extracellular membrane-proximal portion (NC16A domain) of BP180. However, circulating anti-BP230 IgG and IgE autoantibodies are seen in up to 80% and 68%, respectively [26].

Differentiating between IBP and nondrug-induced BP is crucial for effective management. The distinction factors involve timing of onset, distribution of skin lesions, and correlation with immune checkpoint inhibitors. IBP manifests as a generalized pemphigoid-like phenotype affecting the entire body’s skin. In contrast, nondrug-induced BP tends to have a more localized distribution [27,28].

Despite the fact that immune-mediated cutaneous side effects can appear from the first weeks of treatment, the latency period of BP induced by anti-PD1/PD-L1 is usually longer compared to other cutaneous toxicities, ranging from 10 months [29,30,31] to 18 months [32]. Contrary to these typical cases, our patient presented a BP flare at 3 days after receiving treatment, which is an atypical presentation, and it could suggest the presence of some predisposing factors.

The risk factors associated with irAEs have been grouped into three broad categories: (a) patient-specific, based on demographics, social history, and past medical history and drug history; (b) tumor-specific, based on the primary tumor (organ-specific or histology-specific); (c) agent-specific, based on the ICI used, e.g., genetic susceptibility, comorbidities, systemic diseases [33], concomitant use of drugs with known autoimmune toxicity (beta-blockers, antibiotics, diuretics, and nonsteroidal anti-inflammatory drugs (NSAIDs)), and dose-dependent effect of ICIs [34,35]. However, up to this point, no plausible explanation is available for this in the current literature. Chennamadhavuni et al. provide a description stating that biomarkers associated with immune-related adverse events (irAEs) encompass circulating blood counts, cytokines, autoantibodies, HLA genotypes, microRNA, gene expression profiling, and serum proteins. Nevertheless, the efficacy of these markers is not well supported by substantial evidence [36].

An extensive US analysis of data gathered between 2002 and 2012 revealed that BP was unambiguously linked to obesity, hypertension, high glycemic index, and imbalance of lipids in the blood, which suggests a possible link between BP and metabolic syndrome [37,38]. According to a past study, males are slightly more impacted than females. However, up to this point, no plausible explanation is available for this in the current literature. Also, the ailment mainly affects the elderly (65 to 79 years of age) [39,40].

IL-5 is a promising biomarker to reveal disease severity. Eosinophil cationic protein (ECP) causes blister formation, and high levels of ECP and D-dimers in serum indicate BP pathology [41,42]. MiR-1291, a noncoding RNA, may serve as a biomarker for autoimmune diseases. Serum expression of MiR-1291 reflects BP activity, with a recent study showing a significant increase in active individuals (sensitivity: 75.56% and specificity: 81.03%) [43]. These biomarkers are crucial for diagnosing and managing autoimmune diseases.

The recent advent of multiomics, combining different technologies, such as genomics, transcriptomics, proteomics, and metabolomics, has greatly expanded our understanding of the mechanisms involved in irAEs and the ability to predict their occurrence. By analyzing the genome, it becomes possible to identify mutations responsible for ICI resistance and irAEs. This contributes to a better understanding of the underlying mechanisms of irAEs and improves our ability to predict the associated risks. Although, the current state of research on the mechanisms underlying irAEs is still in its infancy, a commonly accepted explanation for irAEs has yet to be found [44].

A possible link may exist between the onset of irAE and the effectiveness of ICIs. Raquel Romão et al. conducted a study in 2023 on 155 tumor patients receiving ICIs. Patients who developed irAE gave a higher objective response rate (ORR) (18.7%), and the risk of death was 33% lesser than patients without irAEs [45]. A recent meta-analysis by Zhao et al. of 789 cases of re-exposure to ICIs suggests that despite the fact that retreatment with ICIs is associated with a higher recurrence of irAEs, no higher number of high-grade irAEs or less efficacy is observed [46]. As there is a little evidence on the safety and effectiveness of ICI retreatment after severe irAE, there is a need for a multidisciplinary assessment as well as the harmonization of consensus guidelines to standardize therapeutic approaches [47].

## 4. Conclusions

The increase in therapeutic indications for ICIs implies that oncologists need to develop more expertise to identify patients at an increased risk of irAEs. To the best of our knowledge, the current case represents the first documented occurrence of an early-onset severe dermatological irAE. In our patient, the convergence of more than one risk factor may have contributed to the early onset of BP (aged patient, obesity, and comorbidities). However, the exceptional nature of this case highlights the need to emphasize the importance of other predisposing factors associated with toxicity. To further maximize the benefit of patients and reduce the risk of toxicity, it is crucial to create predictive indicators for the incidence of irAEs, screen high-risk groups, monitor the change in irAEs, and appraise the result of irAEs. Likewise, there is a need to establish an interdisciplinary consensus for the management of irAEs, as well as the possibility of retreatment.

## Data Availability

Not applicable.
